# Spatiotemporal, kinematic and kinetic assessment of the effects of a foot drop stimulator for home-based rehabilitation of patients with chronic stroke: a randomized clinical trial

**DOI:** 10.1186/s12984-022-01036-0

**Published:** 2022-06-07

**Authors:** Yu Rong Mao, Jiang Li Zhao, Min Jie Bian, Wai Leung Ambrose Lo, Yan Leng, Rui Hao Bian, Dong Feng Huang

**Affiliations:** 1 Department of Rehabilitation Medicine, The Seventh Affiliated Hospital, Sun Yat-Sen University, Shenzhen, China; 2 Guangdong Engineering and Technology Research Center for Rehabilitation Medicine and Translation, Guangdong, China; 3 Department of Rehabilitation Medicine, The First Affiliated Hospital, Sun Yat-Sen University, Guangzhou, China

**Keywords:** Stroke, Peroneal nerve electric stimulate, Motor analysis, Gait training

## Abstract

**Background:**

Gait disability affects the daily lives of patients with stroke in both home and community settings. An abnormal foot–ankle position can cause instability on the supporting surface and negatively affect gait. Our research team explored the ability of a portable peroneal nerve-targeting electrical stimulator to improve gait ability by adjusting the foot–ankle position during walking in patients with chronic stroke undergoing home-based rehabilitation.

**Methods:**

This was a double-blinded, parallel-group randomized controlled trial. Thirty-one patients with chronic stroke and ankle–foot motor impairment were randomized to receive 3 weeks of gait training, which involved using the transcutaneous peroneal nerve stimulator while walking (tPNS group; n = 16, mean age: 52.25 years), or conventional home and/or community gait training therapy (CT group; n = 15, mean age: 54.8 years). Functional assessments were performed before and after the 3-week intervention. The outcome measures included spatiotemporal gait parameters, three-dimensional kinematic and kinetic data on the ankle–foot joint, and a clinical motor and balance function assessment based on the Fugl–Meyer Assessment of Lower Extremity (FMA-LE) and Berg Balance scales (BBS). Additionally, 16 age-matched healthy adults served as a baseline control of three-dimensional gait data for both trial groups.

**Results:**

The FMA-LE and BBS scores improved in both the tPNS groups (p = 0.004 and 0.001, respectively) and CT groups (p = 0.034 and 0.028, respectively) from before to after training. Participants in the tPNS group exhibited significant differences in spatiotemporal gait parameters, including double feet support, stride length, and walking speed of affected side, and the unaffected foot off within a gait cycle after training (p = 0.043, 0.017, 0.001 and 0.010, respectively). Additionally, the tPNS group exhibited significant differences in kinematic parameters, such as the ankle angle at the transverse plane (p = 0.021) and foot progression angle at the frontal plane (p = 0.009) upon initial contact, and the peak ankle joint angle at the transverse plane (p = 0.023) and foot progression angle (FPA) at the frontal and transverse planes (p = 0.032 and 0.046, respectively) during gait cycles after 3 weeks of training.

**Conclusions:**

Use of a portable tPNS device during walking tasks appeared to improve spatiotemporal gait parameters and ankle and foot angles more effectively than conventional home rehabilitation in patients with chronic stroke. Although guidelines for home-based rehabilitation training services and an increasing variety of market devices are available, no evidence for improvement of motor function and balance was superior to conventional rehabilitation.

*Trial registration* Chictr, ChiCTR2000040137. Registered 22 November 2020, https://www.chictr.org.cn/showproj.aspx?proj=64424

## Background

Stroke is one of the five leading causes of disability-adjusted life-years worldwide [1]. In China, the increasing incidence of stroke and decrease in related mortality have led to a rapid increase in the burden of society [2]. Gait dysfunction is common among stroke survivors and represents a major burden, while the community commonly might provide no more than 1-year’s rehabilitation service due to lack of therapists [3, 4]. After stroke, most patients experience abnormal lower extremity movement and a plantarflexion or inversion pattern of hemiplegia in the ankle and foot during the swing phase of gait [5]. Estimates suggest that 20–30% of stroke survivors experience ankle and foot drop and/or inversion, which causes abnormal gait [4]. Ankle–foot drop and inversion are caused by abnormal activation of the musculature in the distal lower limb and result in inefficient foot clearance and foot tremor during the swing phase of gait and a less stable stance. Consequently, stroke patients tend to exhibit a compensatory gait pattern involving the affected side, such as steppage gait, hip hiking, toe walking, and forefoot walking. These pathological deviations reduce the walking speed and increase the risk of fall, thus impeding an individual’s ability to walk efficiently both indoors and outdoors and restricting their participation in many activities of daily life and the community. Therefore, studies on stroke motor recovery have frequently identified inadequate ankle–foot control and stability during walking as a key factor to address when attempting to improve gait dysfunction [6–9].

Approaches such as ankle–foot orthosis (AFO), electrical stimulation (ES), and neuroprosthetic implants are used to treat drop foot in stroke survivors [10–12], and a meta-analysis revealed that these approaches yielded similar results [12]. AFO is a traditional treatment for ankle joint immobilization in the neutral position and can be used to support ankle dorsiflexion during the swing phase to improve gait stability. However, as a mechanical device, an static AFO only restricts ankle movement when it is worn, leading to no improvement of muscle activety,, reduced muscle activity and a restricted ankle range-of-motion higher possibility of falling over the long term [13, 14]. ES, an alternative approach, is widely used to achieve more physiological positioning of the ankle and foot and to improve ankle–foot function, for example neuromuscular electrical stimulation (NMES), functional electrical stimulation (FES) multichannel and neuroprosthetic implants with the development of electronic engineering technology [8, 15–17], but multichannel and implanted devices are not used widely in the clinical settings, which limits their translational use in community and home settings.

Studies conducted in the last 10 years have focused on volitional muscle activation combined with lower motor neuron stimulation, which contributes to several possible mechanisms of neuromuscular plasticity, including repeated muscle contractions leading to increased oxidative capacity; increased numbers of microcapillaries and changes in fiber type at the muscular level; and the convergence of orthodromic or antidromic impulses at the anterior horn, leading to the strengthening of synapses at the spinal level and changes in the cortex [17–19]. These effects of therapy culminate in increased volitional muscle activity in the weak dorsiflexors and evertors of the ankle. Such changes are thought to positively influence other biomechanical features and help restore the associated functions. Several studies have observed sustained improvements in volitional muscles after removal of therapeutic devices, particularly in terms of improved gait capacity during the chronic post-stroke stage [19–21]. One type of FES treatment, the transcutaneous peroneal nerve stimulator (tPNS), involves the placement of electrodes on the skin surface above the peroneal nerve, and based on the mechanisms of effects on volitional muscle activity combined with lower motor neurone stimulation during walking to acquire a more physiological positioning of ankle and foot. This clinically accepted rehabilitation intervention has been demonstrated to be more efficient than AFO, especially in terms of improving walking speed [22, 23]. However, a multicenter prospective randomized study found that the use of peroneal nerve FES was equivalent to device-free gait training in terms of the clinical outcomes of improvements in walking speed, the Fugl–Meyer Assessment of Lower Extremity (FMA-LE) score, ankle muscle strength and dorsiflexion performance [24].

Above studies in different views of the tPNS suggest that this therapeutic device is an appropriate alternative therapeutic device using at chronic stroke survivors for gait training. Other studies have focused on the physiological and biomechanical effects of tPNS on the kinematics and kinetics of the hip, knee, and ankle joints [16, 22, 25]. However, excessive pronation and supination of the foot and hyper-planta/dorsiflexion of the ankle joint directly result in an abnormal and unstable stance and toe clearance during walking, leading to a tilted posture and increasing the fall risk. The evidence so far for improvement of foot activity is still limited, especially its biomechanical analysis. This study focused on quantitative analysis of both kinematics and kinetics of the ankle joint and foot progression angles (FPA) and spatiotemporal gait and clinical assessment parameters. We aimed to determine the efficacy of tPNS among chronic stroke population and its feasibility for use as a rehabilitation training tool in home and community settings. This is important because most discharged stroke patients in China receive inadequate rehabilitation training at home due to a shortage of rehabilitation therapists.

Therefore, in our study, some chronic stroke patients discharged were recruited to randomized into two groups, including the home-based or community-based gait training was arranged via tPNS or self-training without tPNS with therapist’s guidance lasted for 2 h each day during 3 weeks. The results of gait parameters and clinical assessment outcomes were compared between groups before and after interventions.

## Methods

### Participants

All participants of groups with intervention were recruited from patients with stroke who were discharged from the Affiliated Hospital of Sun Yat-Sen University, China. Individuals with foot drop secondary to ischemic stroke who met the inclusion and exclusion criteria were enrolled and randomized to receive either tPNS or conventional therapy (CT). The inclusion criteria were: (1) onset of ischemic stroke ≥ 12 months before enrollment; (2) a positive response to tPNS testing (i.e., ability to achieve passive ankle dorsiflexion) and ability to walk indoors for at least 10 m without an assistance device; (3) adequate ability to understand and communicate, with a Mini-Mental State Examination score ≥ 21; and (4) no history of tPNS use before enrollment. The exclusion criteria were: (1) inability to stabilize the ankle joint or contract the posterior calf while standing without AFO; (2) use of muscle-relaxing drugs, including those administered orally or via lower extremity injection, in the past 3 months; (3) comorbid peripheral neuropathy or vascular disease that would limit ambulation or ES; and (4) other diseases, such as cachexia or skin unsuitable of electrodes (e.g., allergy to adhesive) for participation.

The sample size was calculated based on the outcome of gait speed by meas of G*Power software (Düsseldorf, Germany). The alpha error probability and power were set to 0.05 and 0.8, respectively. The effect size was set to 0.6, according to the similar pevious studies. More than 24 samples were required consequently.

All participants with foot drop and hemiplegia secondary to chronic ischemic stroke were recruited by a specific study team member. Once a participant enrolled, the member got access to a central operating system, which saved the random allocation computed by a statistical expert from Sun Yat-sen University, and assigned particitants according to the numbers of 001 and 002, on behalf of the tPNS group and CT group, respectively. The member who assessed participants was blinded to the intervention allocation and did not take part in other tasks. The trial then stopped due to the amount of enrolled participants exceeding the targeted sample size. All participants received instruction on home-based rehabilitation training, and those in the tPNS group were requested to use the foot drop-stimulating device during daily activity. Additionally, some healthy adults matched to the participants by age, weight, height, leg length, and gender were recruited via advertisement, and their data served as a baseline reference for the three-dimensional analysis of ankle–foot motion. This study was approved by the Human Subjects Ethics Subcommittee of the First Affiliated Hospital, Sun Yat-Sen University, China. Written consent was obtained from all participants before the experiment.

### Foot drop stimulator

**Fig. 1:**
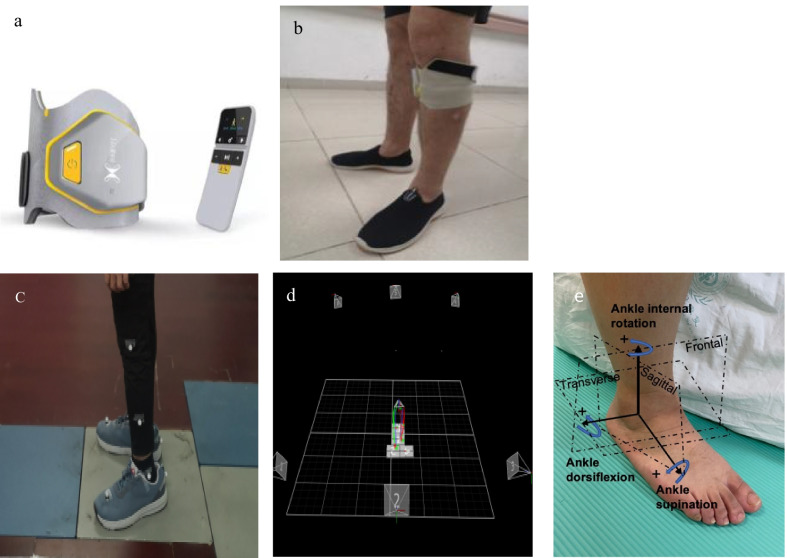
**a** tPNS device, **b** stroke patients wearing the tPNS, **c**, **d** three-dimensional data capture, **e** direction in gait data

The tPNS device (XFT-2001D) was provided by Shenzhen XFT Medical Limited (ISO13485, Shenzhen, China). The single-channel device consisted of a 3.4 V, 480 mAh rechargeable power supply equipped with a USB port and working current of < 90 mA. A wireless remote controller was used to control the switch, select the therapy mode of walking and increase or decrease the dose. Skin surface electrodes were placed at the proximal lateral condyles of the tibia to stimulate the peroneal nerve during the gait cycle. The frequency (17–33 Hz), pulse width (100–300 µs) and intensity of the generated electrical pulse were customized to produce the desired dorsiflexion during gait. The timing of tPNS activation and duration of stimulation were controlled by a tilt sensor and accelerometer according to the position and angle of the calf and speed of oscillation during the gait cycle. The sensor and accelerometric device (73 mm × 70 mm × 10 mm, 43 g) was placed parallel to the surface of ipsilareral tibia just below the knee and properly aligned using anatomical landmarks, visual indicators and dorsiflexion during walking (Fig. 1a).

### Intervention

Participants in the tPNS group were taught to operate the device by a member of the study staff. Each subject was then fitted with a tPNS, the device side of which was consistent with the paralyzed side. A physiotherapist provided exercises to practice following the instructions, placing the electrodes, adjusting the stimulation intensity and taking safety precautions, followed by a 15-min walking period in the corridor outside the laboratory (Fig. 1b). All participants were then given the tPNS device to use when walking at home or in the community, and were asked to wear the device during everyday ambulation training. All participants were instructed to walk at home or in the community while wearing the device for 15–60 min everyday during the first week, > 60 min during the second and the third week wearing as long as possible during daily walking. On the other hand, participants in the CT group received guidance on home-based rehabilitation, including daily walking and simple gait exercises from a motor relearning program, such as straight-line walking, sideway walking, standing with affected-side load, and threshold crossing. Participants in both group actually trained for about 2 h each day throughout the 3-week intervention period, and should send training duration and training condition by means of representative gait-training video via WeChat video to the physiotherapist everyday. Participants were given training suggestions through the physiotherapist via WeChat or were encouraged to visit our lab on working days if they needed additional advice, and the therapist would give advice based on the training performance. The walking activities could be conducted indoors or outdoors and at a self-selected speed based on the participant’s ability.

### Outcome measures

All outcomes were measured before and after the 3-week intervention by the same rehabilitation physician and laboratory researchers using standard operating procedures for groups with intervention. For age-matched healthy adults, they were assessed once they were enrolled. All subjects wore flat shoes and close-fitting pants during the trials. Quantitative gait analysis was performed simultaneously using a Vicon system (VICON MX13, VICON Peak, Oxford, UK) and four AMTI force plates (AMTI, OR6-7, Watertown, MA, USA) with a sampling frequency of 1000 Hz. The analysis included spatiotemporal parameters and kinematic and kinetic data. Six infrared 100-Hz cameras recorded the locations of 16 passive reflective markers taped to the skin over bony landmarks of the pelvis and both lower limbs, following a standard PlugInGait biomechanical model supplied by Vicon Nexus (version 1.7.1). Reflective markers were applied to the skin or close-fitting pants over the anatomical positions of the pelvis, thigh and crus. The marker corresponding to the second metatarsal head position was secured directly to a shoe. The positions of the markers were consistent between the two groups. During the gait capture trials, all subjects were asked to walk forward across the floor for 10 m and return at a self-selected walking speed. The subjects walked without canes, AFOs, or other assistive devices during the trials (Fig. 1c, d). A minimum of 10 trials were conducted for each patient, and six successful gait cycles (defined as one foot on one force plate without delay or pause) were selected for analysis. Clinical outcomes were also measured using the FMA-LE [26] and the Berg Balance Assessment Scale (BBS) [27, 28].

### Data processing and analysis

Polygon (version 3.5.1) from Vicon system was used to process the quantitative three-dimensional gait analysis data from the collected data for above-mentioned successful gait cycles. Capture trails were conducted through combined processing, reconstruct, labeling, and data processing in Vicon system, then export to the Polygon software. In total, representatitve value curves for the six gait cycles were obtained if no missing or incomplete data and evident violation from other cycles. The direction of joint motion is followed by right-handed rule (i.e. ankle joint and FPA in the sagittal plane with + and − are dorsiflexion and planterflexion respectively; the data in the frontal plane with + and − are inversion and eversion respectively; the data in the transverse plane with + and − are adduction and abduction respectively) (Fig. 1e). The spatiotemporal gait parameters and kinematic and kinetic data of the ankle joint and FPA were extracted and analyzed. The spatiotemporal gait parameters were the cadence, stride time and length, step time and length, single and double support time, and walking speed. The ankle and FPA and mean gait cycle for affected limb were measured in three planes; the initial contact (IC), a kinematic parameter, was chosen to determine the stability and positioning of the distal lower extremity in the early stance. The peak angle in each gait cycle was analyzed and used to compare differences in gait performance between the groups in terms of foot–toe clearing and compensation at the proximal lower extremity joint. Of the kinetic parameters, the ankle joint peak moment in the three planes and power were chosen to analyze ankle stability.

The data analysis was performed using SPSS (version 20.0; IBM, Inc., Armonk, NY, USA). The mean values and standard deviations of demographic characteristics (age and sex), the clinical course and anthropometric data (body weight, leg length, knee width and ankle width) at baseline were computed and compared between the two trial groups and healthy adults using a one-way analysis of variance (Table 1). The normality of distribution and equal variance were performed. Then paired samples t-test was used to compare differences within each group from before to after the intervention for normal distribution data, while Mann–Whitney U test was used for violated assumption. Differences between the CT and tPNS groups were identified using the independent samples t-test. The p value of < 0.05 was considered significant. The plotted curves of kinematic data include the average results from six gait cycles.

**Table 1:** Demographic characteristics including means (standard deviation)

Demographics	Normal	tPNS	CT	P
	X (SD)	X (SD)	X (SD)	
Affected side (left number)	–	16 (9)	15 (8)	
Months of post ischemic	–	19.80 (5.86)	19.73 (4.11)	0.973
Sex (female number)	16 (3)	16 (3)	15 (3)	
Age (y)	57.94 (6.88)	52.25 (9.21)	54.80 (10.64)	0.213
Height (mm)	1631.88 (61.67)	1663.13 (74.54)	1648.67 (67.39)	0.143
Body weight (kg)	60.18 (10.59)	67.33 (10.09)	65.49 (10.48)	0.437
Leg length (mm)	825.94 (36.93)	847.81 (50.29)	830.67 (65.27)	0.462
Knee width (mm)	103.13 (8.72)	100.94 (8.41)	100.33 (7.67)	0.615
Ankle width (mm)	71.81 (6.44)	72.81 (5.15)	74.06 (7.25)	0.614

P value for tPNS and CT comparison

## Results

**Fig. 2:**
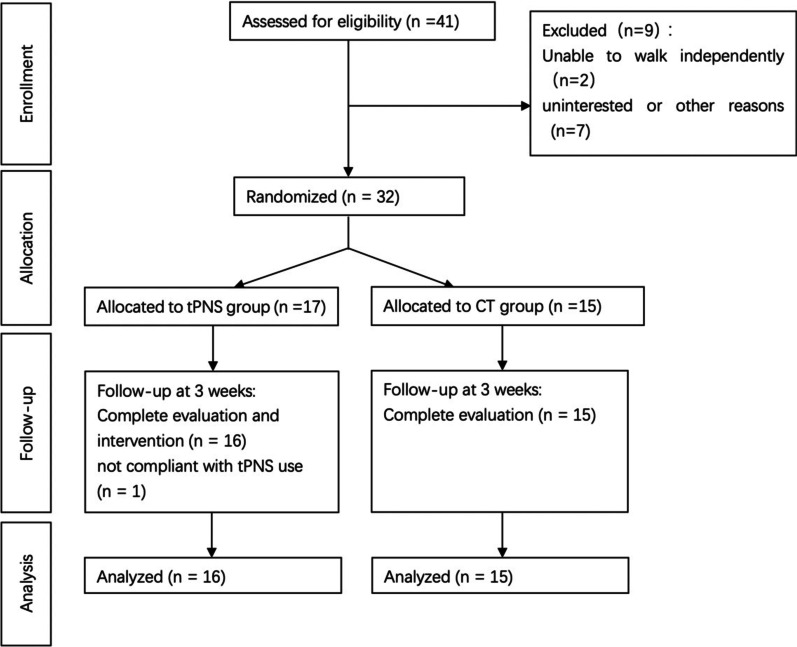
Flow diagram for patients selection process

Thirty-two patients with ischemic stroke who met the inclusion criteria were enrolled. According to the randomisation sequence, 17 participants were randomized to the tPNS group and 15 were randomized to the CT group. One participant was not compliant with tPNS use during daily walking activities. Therefore, 31 participants completed the study and were included in the final data analysis, as shown in Fig. 2. The demographic of the 31 stroke patients and 16 matched healthy adults are shown in Table 1, and a comparison of demographic and anthropometric data and FMA-LE and BBS scores between the CT and tPNS groups before the intervention revealed no statistically significant differences.

### Balance and lower motor function

Compared with the data before the intervention, the data collected after the 3-week period of home- or community-based rehabilitation revealed significant differences in the FMA-LE scores and BBS scores in both the tPNS (p = 0.004 and 0.001, respectively) and CT groups (p = 0.034 and 0.028, respectively). No significant between-group differences in either score were observed after the 3-week training period (see Table 2).

**Table 2:** Motor function of lower extremity and balance for tPNS group and CT group

Demographics	tPNS	CT	Between-group difference
	X (SD)	X (SD)	
FMA-LE (pre)	25.40 (5.21)	26.00 (4.97)	0.706
FMA-LE (post)	26.56 (4.82)*	26.93 (5.26)*	0.967
Berg (pre)	48.47 (4.27)	45.67 (7.23)	0.210
Berg (post)	51.60 (3.68)*	49.47 (4.50)*	0.159

*Significant difference for within group comparison (P < 0.05)

### Spatiotemporal gait parameters

Compared with the measures before training, the tPNS group exhibited significant differences in the gait parameters of opposite foot off, double support, stride length and walking speed after the 3-week training period. Additionally, post-hoc pairwise comparisons revealed significant differences between the tPNS and CT groups in terms of the opposite foot off (%) (11.42 ± 3.87 vs. 17.75 ± 10.13, p = 0.045) and opposite foot contact (%) values (42.21 ± 6.56 vs. 48.70 ± 5.97, p = 0.017). No significant changes in spatiotemporal gait parameters were observed within the CT group from before to after the intervention (see Table 3).

**Table 3:** Spatiotemporal parameters for normal, tPNS, and CT group

Spatiotemporal parameters	Normal	tPNS		P	CT		P
	X (SD)	X (SD)			X (SD)		
		Pre-training	Post-training		Pre-training	Post-training	
Cadence (steps/min)	105.49 (10.34)	71.94 (15.98)	78.44 (17.41)	0.056	76.73 (18.31)	77.72 (18.78)	0.713
Stride time (s)	1.15 (0.11)	1.77 (0.46)	1.63 (0.46)	0.130	1.68 (0.51)	1.68 (0.52)	0.986
Less-affected foot off (%)	10.79 (2.01)	15.47 (7.15)	11.42 (3.87)*	0.010	16.46 (6.32)	17.75 (10.13)	0.634
Less-affected foot contact (%)	49.69 (1.38)	43.89 (5.72)	42.21 (6.56)*	0.221	46.04 (4.75)	48.70 (5.97)	0.163
Step time (s)	0.58 (0.06)	1.01 (0.35)	0.96 (0.37)	0.506	0.92 (0.33)	0.85 (0.24)	0.290
Single support (s)	0.45 (0.04)	0.48 (0.08)	0.48 (0.09)	0.813	0.48 (0.13)	0.49 (0.09)	0.715
Double support (s)	0.26 (0.04)	0.61 (0.36)	0.49 (0.09)	0.043	0.58 (0.36)	0.57 (0.40)	0.904
Foot off (%)	61.34 (1.79)	60.40 (7.45)	58.95 (6.62)	0.071	62.05 (6.18)	62.73 (6.81)	0.430
Stride length (m)	1.07 (0.10)	0.70 (0.29)	0.78 (0.24)	0.017	0.73 (0.26)	0.74 (0.25)	0.607
Step length (m)	0.53 (0.05)	0.37 (0.17)	0.41 (0.11)	0.060	0.37 (0.12)	0.39 (0.12)	0.129
Walking speed (m/s)	0.95 (0.13)	0.44 (0.25)	0.53 (0.23)	0.001	0.49 (0.26)	0.50 (0.24)	0.568

*Significant difference between two groups comparison at post-training (P < 0.05)

### Kinematic parameters

In the tPNS group, significant improvements were observed from before to after training in the average ankle angle in the transverse plane (− 9.32° vs. 2.28°) and FPA in the frontal and transverse planes (− 3.01° vs. 0.92°; − 13.94° vs. − 9.42°, respectively) at the IC. No significant differences in these parameters were observed within the CT group (see Table 4). Similarly, only the tPNS group exhibited significant improvements from before to after training in the average ankle transverse peak during the stance and swing phases (− 0.40° vs. 11.86°; − 26.29° vs. − 14.48°, respectively) and in the maximum foot progression peak in the frontal and transverse planes (2.71° vs. 6.13°; − 9.34° vs. − 4.27°, respectively). A statistically significant difference in the maximum foot progression peak in the sagittal plane was observed between the tPNS and CT groups after the intervention (see Table 5).

**Table 4:** Kinematic result with three groups at three planes

Kinematic parameters (°)	Normal	tPNS		P	CT		P
	X (SD)	X (SD)			X (SD)		
		Pre-training	Post-training		Pre-training	Post-training	
Ankle IC							
Sagittal	− 7.12 (5.18)	− 4.45 (5.64)	− 2.94 (7.71)	0.364	− 3.77 (6.87)	− 3.29 (4.68)	0.795
Frontal	− 0.17 (1.11)	0.83 (3.51)	− 0.64 (2.95)	0.195	2.08 (3.38)	0.70 (4.30)	0.097
Transverse	2.21 (12.86)	− 9.32 (17.08)	2.28 (11.02)	0.021	− 14.23 (17.29)	− 9.14 (23.91)	0.345
Foot IC							
Sagittal	− 93.46 (6.02)	− 89.11 (9.51)	− 83.69 (21.16)	0.291	− 91.64 (5.12)	− 90.82 (6.80)	0.731
Frontal	4.47 (3.64)	− 3.01 (6.46)	0.92 (4.21)	0.009	− 1.76 (4.88)	− 4.98 (17.51)	0.531
Transverse	− 11.37 (5.98)	− 13.94 (9.90)	− 9.42 (5.48)	0.050	− 14.57 (9.65)	− 14.75 (9.37)	0.888

P value for within group comparison

**Table 5:** Ankle and foot progress angle peak at stance phase and swing phase

Kinematic parameters (°)	Normal	tPNS		P	CT		P
	X (SD)	X (SD)			X (SD)		
		Pre-training	Post-training		Pre-training	Post-training	
Ankle StP_max_							
Sagittal	9.30 (4.62)	12.29 (7.57)	14.57 (6.87)	0.134	13.11 (5.88)	14.88 (3.54)	0.264
Frontal	1.66 (2.04)	5.20 (4.90)	2.80 (4.00)	0.132	4.35 (4.04)	3.20 (4.21)	0.150
Transverse	9.20 (11.73)	− 0.40 (18.12)	11.86 (11.76)	0.023	− 5.26 (16.19)	0.15 (24.85)	0.334
Ankle SwP_min_							
Sagittal	− 17.17 (7.03)	− 8.76 (5.74)	− 5.91 (7.95)	0.113	− 8.14 (7.80)	− 6.58 (5.08)	0.452
Frontal	− 0.93 (1.24)	− 1.14 (4.28)	− 2.61 (3.05)	0.283	0.66 (2.89)	− 1.17 (5.00)	0.066
Transverse	− 14.16 (12.49)	− 26.29 (18.86)	− 14.48 (15.09)	0.039	− 26.51 (15.95)	− 21.09 (22.03)	0.258
Foot progress StP_max_							
Sagittal	− 20.15 (8.90)	− 53.32 (16.08)	− 66.84 (25.59)*	0.101	− 49.18 (15.13)	− 48.06 (17.57)	0.695
Frontal	8.25 (3.72)	2.71 (6.03)	6.13 (3.75)	0.032	4.64 (3.92)	2.00 (16.42)	0.605
Transverse	− 3.73 (6.01)	− 9.34 (10.44)	− 4.27 (5.66)	0.046	− 8.38 (7.97)	− 9.28 (8.72)	0.523
Foot progress SwP_min_							
Sagittal	− 101.56 (6.74)	− 99.58 (6.04)	− 90.79 (20.06)	0.148	**− 99.56 (11.77)**	**− 97.28 (7.90)**	**0.806**
Frontal	− 1.43 (2.56)	− 9.31 (6.83)	− 7.35 (5.31)	0.211	− 8.13 (6.34)	− 9.57 (17.97)	0.771
Transverse	− 18.76 (6.25)	− 26.01 (13.70)	− 22.20 (8.07)	0.247	− 25.27 (11.21)	25.65 (12.42)	0.858

StP_max_: maximum value in the stance phase; SwP_min_: minimum value in the swing phase

The data with bold: not normally distributed with Mann–Whitney U test

P value for within group comparison; *significant difference between two groups comparison at post-training (P < 0.05)

**Fig. 3:**
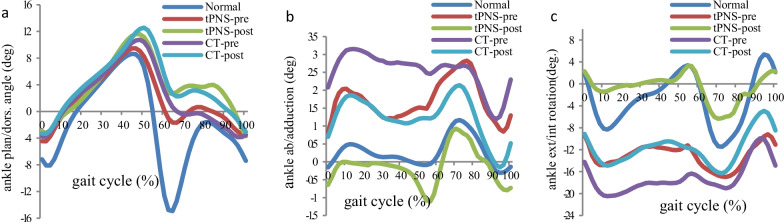
Mean ankle joint kinematic curve for all participants, **a** sagittal plane, **b** frontal plane, **c** transverse plane

**Fig. 4:**
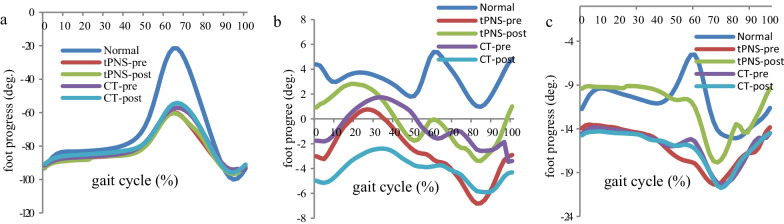
Mean foot progress kinematic curve for all participants, **a** sagittal plane, **b** frontal plane, **c** transverse plane

Figures 3 and 4 show the average kinematic trajectories of the ankle joint and foot progression in three planes as measured in healthy adults and participants with stroke in the tPNS and CT groups. Using the healthy adults as a baseline reference, participants in the tPNS group exhibited marked improvements in the average kinematic trajectories in the frontal and transverse planes after the intervention.

### Kinetic

**Fig. 5:**
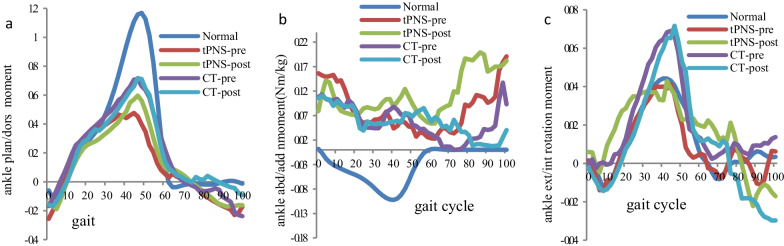
Mean ankle join kinetic curve for all participants, **a** sagittal plane, **b** frontal plane, **c** transverse plane

**Fig. 6:**
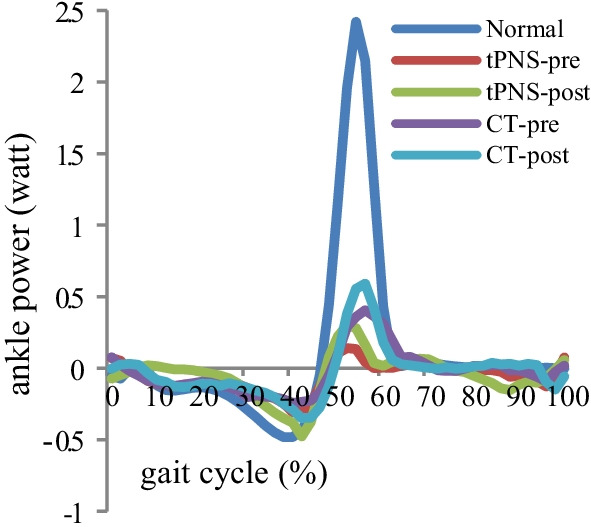
Mean ankle joint power for all participants

No significant within-group differences were observed in the moment peak and ankle power peak in any plane after the intervention. However, a significant between-group difference was observed in the maximum ankle joint peak in the sagittal plane after the intervention (see Table 6). Figures 5 and 6 show the average moment and power trajectories of the ankle joint as measured in healthy adults and participants with stroke in the tPNS and CT groups, and both groups showed a significant decresing sagittal moment and power peak compare to healthy group.

**Table 6:** Ankle moment peak at stance phase and swing phase and power peak at stance phase

Kinetic parameters(N·m/kg, W/kg))	Normal	tPNS		P	CT		P
	X (SD)	X (SD)			X (SD)		
		Pre-training	Post-training		Pre-training	Post-training	
Ankle StP_max_							
Sagittal	1.20 (0.28)	0.73 (0.27)	0.80 (0.36)*	0.460	0.97 (0.57)	1.01 (0.32)	0.783
Frontal	0.10 (0.10)	**0.51 (0.38)**	**0.55 (0.40)**	**0.653**	**0.44 (0.45)**	**0.42 (0.42)**	**0.512**
Transverse	0.09 (0.05)	0.11 (0.05)	0.13 (0.08)	0.372	**0.13 (0.08)**	**0.11 (0.09)**	**0.250**
Ankle SwP_min_							
Sagittal	− 0.15 (0.09)	− 0.54 (0.46)	− 0.45 (0.32)	0.478	− 0.56 (0.50)	− 0.43 (0.53)	0.165
Frontal	− 0.18 (0.12)	− 0.08 (0.09)	− 0.05 (0.06)	0.257	− 0.10 (0.13)	− 0.16 (0.210	0.323
Transverse	− 0.06 (0.05)	− 0.08 (0.05)	− 0.08 (0.06)	0.844	0.06 (0.05)	− 0.08 (0.11)	0.394
Ankle power							
StP_max_	2.76 (0.94)	0.61 (0.46)	0.74 (0.55)	0.335	0.97 (0.78)	0.92 (0.61)	0.722
StP_min_	− 0.70 (0.28)	− 0.78 (0.52)	− 0.85 (0.66)	0.699	**− 0.61 (0.57)**	**− 0.68 (0.34)**	**0.233**

StP_max_: maximum value in the stance phase; SwP_min_: minimum value in the swing phase; StP_min_: minimum value in the stance phase

The data with bold: not normally distributed with Mann–Whitney U test

P value for within group comparison; *significant difference between two groups comparison at post-training (P < 0.05)

## Discussion

This study aimed to explore the efficacy and safety of tPNS for facilitating physiological ankle and foot movement in a therapeutic setting, using changes in the participants’ mechanistic outcomes and kinematic and kinetic parameters. We measured and analyzed the spatiotemporal gait parameters, ankle–foot joint angle in three planes and ankle joint moment and power using a three-dimensional motion system in patients with chronic stroke who used a single-channel ES device with a surface electrode over the peroneal nerve in home and community settings for 3 weeks, and observed changes in their clinical motor and balance function. The results demonstrate that tPNS induced changes in these parameters from before to after the training period.

### Clinical outcome of balance and lower motor function

Studies have shown that a 4-week intervention in which foot drop stimulation is delivered via a peroneal nerve stimulation device did not lead to significant changes in the FMA-LE scores of patients with chronic stroke patients relative to the scores of patients receiving CT [22, 29]. A study and systematic review both observed a significant increase in the BBS score but not the FMA-LE score [30, 31]. In contrast, our study showed an improvement in both the FMA-LE and BBS scores within both groups from before to after training, but no significant difference between the tPNS and CT groups. This outcome may be due to the use of valid gait training individualized to our study participants’ daily living environments and their good compliance with either the tPNS or CT protocol, as the consideration of these patients from previous experimental subjects.

### Spatiotemporal gait parameters

Foot drop causes an inefficient gait with asymmetrical parameters. Gait speed is an important factor in an individual’s ambulation status and level of disability, and it has a significant impact on the quality of life of stroke survivors, who must expend a large amount of energy to achieve a slow gait speed [25, 32, 33]. Accordingly, increasing gait speed is the most frequently stated therapeutic goal, especially for community-dwelling individuals [11]. Our observation of a marked improvement in the average walking speed of the tPNS group is consistent with the outcomes observed by Sheffler et al. and Bethoux et al. in studies of patients with chronic stroke [20, 21]. In our study, participants in the tPNS group also exhibited significant improvements in the gait parameters of opposite foot off, double support, and stride length after the 3-week training period, and these changes indicated a shift toward improved health. Other gait parameters, such as cadence, step length, stride time, and step time, also improved after the intervention, although these changes were not significant. After a stroke, hemiplegic patients with foot drop tend to have a slower gait and to take more and shorter steps within the same distance than healthy subjects. Patients with stroke also need more time during the double support phase of the gait cycle to adjust their bodies and maintain balance. The increased walking speed in our study may be due to improvements in the mutual effects of improvements in other parameters. We note that although we observed no significant differences in spatiotemporal gait parameters within the CT group over time, seven of the fifteen participants exhibited declines in these parameters.

### Kinematic and kinetic parameters

The tPNS is used with the intent to increase dorsiflexion during the IC and swing phases and pronation and supination of foot progress in the gait cycle. Our study focused on ankle–foot kinematic and kinetic parameters in three planes and the effectiveness of tPNS device for the rehabilitation of patients with stroke in a home-based environment. During a gait cycle, the IC signals the leg to prepare for the loading response [22]. We observed significant differences in the average IC of the ankle and in the FPA in both the transverse and frontal planes, but did not observe significant differences in the sagittal plane for either the ankle or the foot. The definition of foot progression angle is the angle between the line joining the centre of the ankle joint to the second metatarsal head and the progression axis of the walk. A deviation in FPA has been observed to shift center of pressure mediolaterally [34], so our result may suggest better balance ability for affected limb. The results of our ankle dorsiflexion performance analysis were not in accordance with those of implantable peroneus nerve stimulator and FES in previous studies[15, 16, 25, 35]. For example, Lynne et al. showed a decrease in ankle dorsiflexion during the swing phase after a 12-week course of peroneal nerve stimulation [36]. Our study showed an increasing curve peak during the swing phase. This may have occurred in our study participants from discharge patients, despite the lack of innervation and training of the eversion muscle, FES acted on and trained the dorsiflexion tibialis anterior muscle in the hospital stage in accordance with the overstimulation principal, leading to introversion of ankle joint during walking. In our study, the tPNS device mainly stimulate lower lateral side of the fibula capitula, in order to correct over introversion and dorsiflexion of ankle joint. A lack of dorsiflexion can also result in a secondary malposition, wherein the patient prefers to lift the pelvis and lower extremity on the affected side to step forward, causing a circumduction gait pattern with ankle–foot abduction at the IC through the heel or the full foot. The tPNS device stimulates the peroneal nerve through surface skin electrodes to produce contraction of the associated muscles (musculi tibialis anterior, peronei, extensor digitorum longus and extensor hallucis longus), inducing a change in the ankle–foot joint position during the stance and swing phases. Similar results were also observed in the ankle and FPA peak in three planes during the stance and swing phases. Finally, our study attempted to explore whether tPNS or conventional home-based rehabilitation affects the moment and power of the ankle joint and the moment of foot progress. No significant differences in these parameters were observed between the tPNS and CT groups in this study after the 3-week training period. Possibly, the intervention was too short to demonstrate an effect; however, the short duration of training was chosen with consideration of medical insurance availability. Studies have explained that long-term tPNS usage improves gait performance through the central and peripheral neural mechanisms of increasing oxidative capacity and microcapillary number; at the muscle level, it leads to a restoration of the fiber type, increasing muscular strength and kinematic and kinetic gait parameters in patients after stroke [17, 19, 22]. A 3-week training period may not be sufficient to induce positive changes in the weak dorsiflexors and evertors of the ankle, which would explain the lack of significant differences in moment and power. Our study provided the evidence for application of tPNS in improvement of ankle joint foot progress motion in transverve plane.

## Limitations

This study has some limitations of note. First, the duration of the therapy was fairly short, lack of follow-up after therapeutic effect and a large sample size, which may lead to no significant difference in the intervention group. Second, we only recorded some kinematic and kinetic parameters but did not obtain electromyography recordings of the lower limb muscles, dynamometric measures of objective muscle strength or functional brain imaging data. Finally, the study staff could not determine that the patients were compliant with the instructions to wear the device, and could hardly supervise the real duration and effectiveness of training. Therefore, future studies should increase the length of the intervention; include additional assessments such as electromyography, muscle strength measurement and brain function analyses; and recruit a larger sample for a study with a randomized controlled design. Such changes might enable a better analysis and more detailed understanding of the outcomes and reveal the true effectiveness of tPNS for the rehabilitation of patients with stroke in terms of the ankle–foot drop and inversion.

## Conclusion

The results of this study suggest that tPNS training and conventional home-based rehabilitation are equally effective for improving clinical motor and balance in patients with chronic stroke. However, tPNS appears to have superior effects on spatiotemporal gait parameters and the ankle–foot angle. These results demonstrate the promise of tPNS, and this information may help to enhance guidelines for home-based rehabilitation training services. It may also enable patients or their family members to choose a suitable commercially available device for rehabilitation. However, future studies in this area should include a larger sample and longer duration of intervention.

## Data Availability

The datasets used and/or analyzed during this study are available from the corresponding author on reasonable request.

## Abbreviations

AFO: Ankle–foot orthosis
ES: Electrical stimulation
NMES: Neuromuscular electrical stimulation
FES: Functional electrical stimulation
tPNS: Transcutaneous peroneal nerve stimulator
FMA-LE: Fugl–Meyer Assessment of Lower Extremity
FPA: Foot progression angle
CT: Conventional therapy
BBS: Berg Balance Assessment Scale
IC: Initial contact

## References

[1] Kyu HH (2018). Global, regional, and national disability-adjusted life-years (DALYs) for 359 diseases and injuries and healthy life expectancy (HALE) for 195 countries and territories, 1990–2017: a systematic analysis for the global burden of disease study 2017. Lancet.

[2] Wang Y (2020). Secular trends of stroke incidence and mortality in China, 1990 to 2016: the global burden of disease study 2016. J Stroke Cerebrovasc Dis.

[3] Magdon-Ismail Z (2018). Factors associated with 1-year mortality after discharge for acute stroke: what matters?. Top Stroke Rehabil.

[4] Jorgensen HS (1995). Recovery of walking function in stroke patients: the Copenhagen stroke study. Arch Phys Med Rehabil.

[5] Murray CJ (2012). Disability-adjusted life years (DALYs) for 291 diseases and injuries in 21 regions, 1990–2010: a systematic analysis for the global burden of disease study 2010. Lancet.

[6] Lin PY (2006). The relation between ankle impairments and gait velocity and symmetry in people with stroke. Arch Phys Med Rehabil.

[7] Brandstater ME (1983). Hemiplegic gait: analysis of temporal variables. Arch Phys Med Rehabil.

[8] Yang Y (2018). Effects of neuromuscular electrical stimulation on gait performance in chronic stroke with inadequate ankle control—a randomized controlled trial. PLoS ONE.

[9] Patterson KK (2010). Evaluation of gait symmetry after stroke: a comparison of current methods and recommendations for standardization. Gait Posture.

[10] Mendes LA (2020). Motor neuroprosthesis for promoting recovery of function after stroke. Cochrane Database Syst Rev.

[11] Winstein CJ (2016). Guidelines for adult stroke rehabilitation and recovery. Stroke.

[12] Prenton S (2018). Functional electrical stimulation and ankle foot orthoses provide equivalent therapeutic effects on foot drop: a meta-analysis providing direction for future research. J Rehabil Med.

[13] Mulroy SJ (2010). Effect of AFO design on walking after stroke. Prosthet Orthot Int.

[14] Nikamp C, Buurke J, Schaake L, van der Palen J, Rietman J, Hermens H (2019). Effect of long-term use of ankle-foot orthoses on tibialis anterior muscle electromyography in patients with sub-acute stroke: a randomized controlled trial. J Rehabil Med.

[15] Daniilidis K (2017). Does a foot-drop implant improve kinetic and kinematic parameters in the foot and ankle?. Arch Orthop Trauma Surg.

[16] Kottink AIR (2012). Effects of an implantable two-channel peroneal nerve stimulator versus conventional walking device on spatiotemporal parameters and kinematics of hemiparetic gait. J Rehabil Med.

[17] Everaert DG (2010). Does functional electrical stimulation for foot drop strengthen corticospinal connections?. Neurorehabil Neural Repair.

[18] Chipchase LS, Schabrun SM, Hodges PW (2011). Peripheral electrical stimulation to induce cortical plasticity: a systematic review of stimulus parameters. Clin Neurophysiol.

[19] Gandolla M (2016). The neural correlates of long-term carryover following functional electrical stimulation for stroke. Neural Plast.

[20] Liberson WT (1961). Functional electrotherapy: stimulation of the peroneal nerve synchronized with the swing phase of the gait of hemiplegic patients. Arch Phys Med Rehabil.

[21] Pereira S (2014). Functional electrical stimulation for improving gait in persons with chronic stroke. Top Stroke Rehabil.

[22] Sheffler LR (2013). Spatiotemporal, kinematic, and kinetic effects of a peroneal nerve stimulator versus an ankle foot orthosis in hemiparetic gait. Neurorehabil Neural Repair.

[23] Bethoux F (2015). Long-term follow-up to a randomized controlled trial comparing peroneal nerve functional electrical stimulation to an ankle foot orthosis for patients with chronic stroke. Neurorehabil Neural Repair.

[24] Hachisuka K (2021). Clinical effectiveness of peroneal nerve functional electrical stimulation in chronic stroke patients with hemiplegia (PLEASURE): a multicentre, prospective, randomised controlled trial. Clin Rehabil.

[25] Jaqueline Da Cunha M (2021). Functional electrical stimulation of the peroneal nerve improves post-stroke gait speed when combined with physiotherapy. A systematic review and meta-analysis. Ann Phys Rehabil Med.

[26] Fugl-Meyer AR (1975). The post-stroke hemiplegic patient. 1. A method for evaluation of physical performance. Scand J Rehabil Med.

[27] Berg KO (1992). Clinical and laboratory measures of postural balance in an elderly population. Arch Phys Med Rehabil.

[28] Berg KO (1992). Measuring balance in the elderly: validation of an instrument. Can J Public Health.

[29] Kluding PM (2013). Foot drop stimulation versus ankle foot orthosis after stroke. Stroke.

[30] Bethoux F (2014). The effects of peroneal nerve functional electrical stimulation versus ankle–foot orthosis in patients with chronic stroke. Neurorehabil Neural Repair.

[31] Dunning K (2015). Peroneal stimulation for foot drop after stroke. Am J Phys Med Rehabil.

[32] Kramer S (2016). Energy expenditure and cost during walking after stroke: a systematic review. Arch Phys Med Rehabil.

[33] Beyaert C, Vasa R, Frykberg GE (2015). Gait post-stroke: pathophysiology and rehabilitation strategies. Neurophysiologie clinique = Clin Neurophysiol.

[34] van den Noort J, Schaffers I, Snijders J (2013). The effectiveness of voluntary modifications of gait pattern to reduce the knee adduction moment. Hum Mov Sci.

[35] Lee Y (2014). Functional electrical stimulation to ankle dorsiflexor and plantarflexor using single foot switch in patients with hemiplegia from hemorrhagic stroke. Ann Rehabil Med.

[36] Sheffler LR (2015). Surface peroneal nerve stimulation in lower limb hemiparesis. Am J Phys Med Rehabil.

